# Une cause rare de compression médullaire: kyste arachnoïdien épidural rachidien (à propos de 03 cas)

**DOI:** 10.11604/pamj.2017.26.132.8548

**Published:** 2017-03-09

**Authors:** Abderrazzak El Saqui, Mohamed Aggouri, Mohamed Benzagmout, Khalid Chakour, Mohamed El Faiz Chaoui

**Affiliations:** 1 Service Neurochirurgie, CHU Hassan II, Fès, Maroc

**Keywords:** Kyste arachnoïdien épidural, rachis, IRM, chirurgie, Epidural arachnoid cyst, spine, MRI, surgery

## Abstract

Le kyste arachnoïdien épidural rachidien (KAER) est une affection bénigne, de physiopathologie encore incertaine. Il est le plus souvent asymptomatique mais peut causer des séquelles neurologiques graves surtout quand le traitement n'est pas instauré à temps. Nous rapportons l'expérience du service de Neurochirurgie CHU Hassan II- Fès concernant la prise en charge du KAER à travers l'analyse rétrospective de trois cas. Il s'agit de deux patients de sexe masculin et d'une femme, d'âge moyen de 35 ans (Extrêmes: 16 et 56 ans), tous admis pour un tableau de compression médullaire lente. Tous nos patients ont bénéficié d'une IRM médullaire qui a mis en évidence une collection liquidienne de siège épidural, ayant le même signal que le LCR, comprimant la moelle en regard. Le siège de la collection était thoracique dans tous les cas. Tous nos patients ont été opérés par voie postérieure avec exérèse du kyste et ligature du collet dans deux cas et une plastie durale dans un seul cas. L'étude anatomopathologique a conclu en un kyste arachnoïdien. L'évolution postopératoire était favorable dans tous les cas. Ce travail a comme objectif de mettre le point sur cette pathologie tout en insistant sur la nécessité d'une prise en charge précoce, vu la tendance vers l'aggravation progressive en l'absence de thérapie adaptée et rappeler les particularités cliniques, paracliniques et thérapeutiques de cette affection.

## Abstract (en)

Spinal epidural arachnoid cyst (SEAC) is a benign condition whose pathophysiology is still uncertain. It is most commonly asymptomatic but it can cause severe neurological sequelae especially when treatment is not received in time. We conducted a retrospective study of three patients treated for SEAC conducted in the Neurosurgery Department, Hassan II University Hospital, Fez. We report the case of two male patients and a woman, with an average age of 35 years (range: 16-56 years), admitted with slow progressive spinal cord compression. All patients underwent spinal cord MRI showing epidural fluid collection, having the same signal as that of cerebrospinal fluid, compressing the opposite marrow. The collection was located in the chest in all cases. All patients underwent surgery via posterior approach for cyst resection and cyst neck ligation in two cases and dural plasty in a single case. Anatomo-pathologic examination showed arachnoid cysts. Postoperative outcome was simple in all cases. This study aims to update the current understanding of this pathology while insisting on the need for early management given its tendency toward gradual worsening in the absence of adapt therapy. It also aims to review the clinical, paraclinical and therapeutic features of this condition.

## Introduction

Les kystes arachnoïdiens correspondent à des formations arachnoïdiennes dont les parois sont arachnoïdiennes et ne se différencient pas du tissu arachnoïdien avoisinant. Ils peuvent se développer partout où il existe de l'arachnoïde, avec une tendance à se localiser au niveau des citernes, mais la localisation rachidienne demeure rare. Ces kystes contiennent du LCR de même composition que le LCR voisin, et communique avec les lacs arachnoïdiens jointifs permettant un échange régulier du liquide intrakystique. Le kyste arachnoïdien épidural rachidien (KAER) est une cause rare de compression médullaire. La localisation thoracique est la plus fréquente (65%), mais les autres localisations sont aussi rapportées : la région lombosacrée (13%), thoraco-lombaire (12%), sacrée (7%) et la région cervicale (3%). Le KAER se développe à partir d'une protrusion de l'arachnoïde à travers un défect de la dure mère. Le kyste a un collet en communication avec l'espace sous arachnoïdien spinal et contient du LCR. La cause de ces kystes n'est pas encore définitivement déterminée, mais fort probablement ils ont une origine congénitale ou acquise suite à un traumatisme, une infection ou une inflammation. L'imagerie par résonance magnétique (IRM) reste le moyen d'investigation idéal pour détecter la masse kystique, son étendue, sa topographie et son signal caractéristique similaire à celui du LCR. Le traitement des KAER est chirurgical si le patient devient symptomatique; ce traitement vise à réaliser l'exérèse totale du kyste et, si possible, la ligature du collet.

## Patient et observation

Nous rapportons ci-après l'expérience du service de Neurochirurgie CHU Hassan II- Fès concernant la prise en charge du KAER à travers l'analyse rétrospective de trois cas. Il s'agit de deux patients de sexe masculin et d'une femme, d'âge moyen de 35 ans (Extrêmes : 16 et 56 ans), tous admis pour un tableau de compression médullaire lente. Tous nos patients ont bénéficiés d'une IRM médullaire qui a mis en évidence une collection liquidienne de siège épidural, ayant le même signal que le LCR, comprimant la moelle en regard. Le siège de la collection était thoracique dans tous les cas. Tous nos patients ont été opérés par voie postérieure avec exérèse du kyste et ligature du collet. L'étude anatomopathologique a conclu en un kyste arachnoïdien. L'évolution postopératoire était favorable dans tous les cas. La rééducation fonctionnelle a été un complément thérapeutique systématique chez tous nos patients.

### Observation N° 1

Il s'agit d'un patient âgé de 56 ans, sans antécédents pathologiques notables, qui présentait depuis 04 ans des troubles de la marche avec des névralgies intercostales évoluant dans un contexte d'apyrexie et de conservation de l'état général. L'examen clinique avait retrouvé une discrèteparaparésie grade D de Frankel avec des signes d'irritation pyramidale aux deux membres inférieurs, sans troubles génito-sphinctériens ni troubles sensitifs associés. Le patient rapporte avoir fait une première IRM rachidienne qui avait montré une lésion rachidienne et une intervention chirurgicale lui a été proposée. Cependant, le malade l'avait refusée à l'époque puis fut perdu de vue avant de se présenter aux urgences 03 ans plus tard pour une aggravation de la lourdeur des deux membres inférieurs et apparition de troubles phinctériens à type de fuites urinaires. L'examen à l'admission avait noté une paraparésiespastique lourde (grade C de Frankel), ne permettant plus la marche, associée à une hypoesthésie superficielle à niveau supérieur xiphoïdien. L'IRM médullaire a objectivé un processus de signal comparable à celui du LCR, hypointense en T1 et hyperintense en T2, ne prenant pas le contraste, étendu de T7 à T9, de siège postérieur, refoulant en avant la moelle devenue laminée et plaquée contre le mur postérieur des corps vertébraux. L'angle de raccordement ouvert entre le kyste et la dure-mère, mis en évidence grâce à l'aspect triangulaire de la graisse épidurale au niveau des deux pôles de la lésion, a d'emblée fait suspecté un kyste extradural.

### Observation N° 2

Notre deuxième patiente est Mademoiselle B.A âgée de 35 ans, ayant comme antécédent une néphrectomie gauche faite en 2004 avec à l'histologie un carcinome à cellules claires grade II de Fuhrman. La patiente a aussi été opérée pour un fibrome utérin en 2009 où elle a bénéficié d'une myomectomie. Le début de la symptomatologie clinique remontait à 03 ans avant son admission par l'apparition de rachialgies augmentant progressivement d’intensité, répondant au traitement symptomatique à base d'antalgiques et d'antiinflammatoires. La patiente rapporte aussi qu'un an avant son admission, elle a commencé à avoir des troubles de la marche à type de claudication intermittente avec un périmètre de marche de plus en plus réduit, associé à des sciatalgies bilatérales mal systématisées prédominant à gauche et des troubles sphinctériens à type d'impériosité mictionnelle. A l'admission, la patiente était consciente (GCS à 15), stable sur le plan hémodynamique et respiratoire, apyrétique, ayant des conjonctives normocolorées. L'examen neurologique avait objectivé une paraparésie grade D de Frankel avec des signes d'irritation pyramidale (ROT vifs, Babinski positif à gauche, trépidation épileptoïde du pied gauche, clonus bilatéral de la rotule). L'exploration de la sensibilité était sans anomalies. Devant ce tableau clinique suggestif d'une compression médullaire lente, une IRM médullaire a été réalisée. Cette dernière a objectivé une lésion intrarachidienne extradurale postérieure, à hauteur de D7, bien limitée, mesurant 35mm de hauteur, et 10mm d'épaisseur, de signal liquidien (hypointense T1, hyperintense T2), ne se rehaussant pas après injection du produit de contraste, partiellement engagée dans le foramen D7-D8 droit.

### Observation N° 3

**Figure 1:**
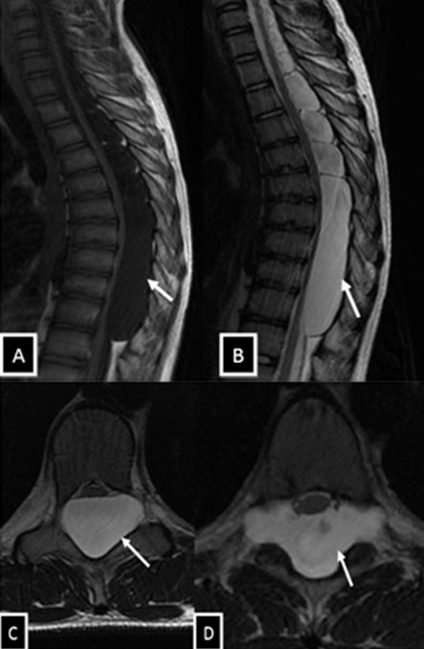
IRM médullaire en coupes sagittales T1 avec contraste (A), T2 (B), et axiales T2 (C, D) montrant une formation kystique cervico-dorsal, de signal comparable à celui du LCR, hypointense en T1 et hyperintense en T2, ne se rehaussant pas après injection de contraste, de siège postérieur, plaquant la moelle contre le mur postérieur des corps vertébraux

**Figure 2:**
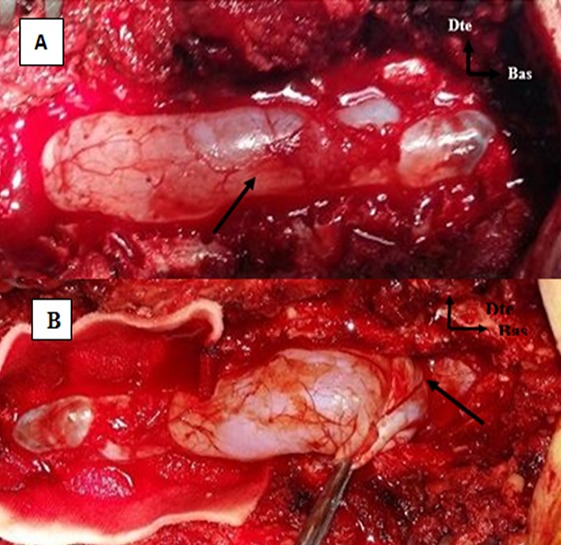
(A) aspect per opératoire après réalisation d'une laminectomie: le kyste arachnoïdien est formé d'une membrane fine translucide renfermant du LCR (Flèche), (B) Début de la dissection du kyste par rapport au plan dural (Flèche)

**Figure 3:**
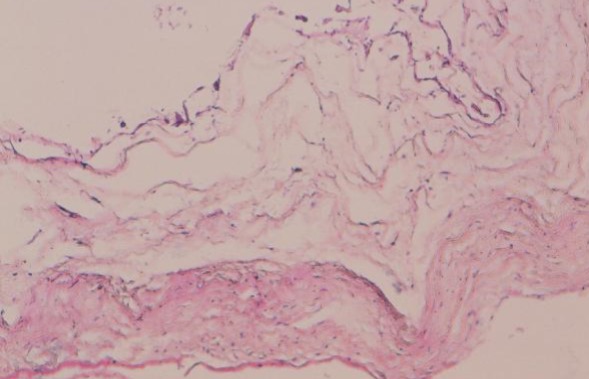
Image microscopique de la paroi du kyste montrant une paroi fibreuse tapissée par un revêtement arachnoïdien fait de cellules au cytoplasme abondant éosinophile et au noyau régulier (HES x 10)

Il s'agit de l'enfant D.M âgé de 14 ans, sans antécédents pathologiques notables, qui présentait depuis 02 mois une lourdeur des deux membres inférieurs, sans notion de douleur rachidienne ni autre signe associé; le tout évoluant dans un contexte d'apyrexie et de conservation de l'état général. A l'admission, l'examen clinique avait retrouvé une paraparésie grade C de Frankel avec un syndrome pyramidal aux deux membres inférieurs (ROT vifs, Babinski bilatéral), sans troubles génito-sphinctériens ni troubles sensitifs associés. Devant ce tableau clinique évoquant une compression médullaire lente, une IRM médullaire a été réalisée. Cette dernière a objectivé une formation kystique de signal comparable à celui du LCR, hypointense en T1 et hyperintense en T2, ne se rehaussant pas après injection du produit de contraste, étendu de C6 à T10, de siège postérieur, refoulant la moelle épinière en avant contre le mur postérieur des corps vertébraux (Figure 1). Le patient a été opéré par voie postérieure. La réalisation d'une laminectomie de T5 à T10 a permis de découvrir un processus extradural, à paroi fine translucide, de contenu liquidien. La dissection du kyste par rapport au plan dural a été périlleuse: la paroi durale était très amincie par endroits. La paroi du kyste a été rompue laissant écouler un liquide eau de roche, d'aspect similaire à celui du LCR. La dissection de la paroi du kyste a laissé découvrir une large déhiscence à la face postérieure de la dure mère étendue de T7 à T9. Après exérèse totale du kyste, cette déhiscence a été réparée au moyen d'une plastie aponévrotique (Figure 2). Durant la période d'hospitalisation postopératoire, une nette récupération motrice a été constatée. L'examen anatomopathologique a confirmé la nature arachnoïdienne de la lésion (Figure 3).

## Discussion

Habituellement, la paroi d´un kyste arachnoïdien épidural rachidien (KAER) est constituée d'un tissu conjonctif fibreux avec un alignement d´arachnoïde monocellulaire intérieur; cependant, cet alignement est parfois absent à l´examen histologique. Une classification des kystes arachnoïdiens en fonction de la localisation, du siège et de la compression nerveuse qu'ils entrainent a été proposée pour la première fois en 1988 par Nabors MW et al. [1]. Cette classification distingue trois types de kystes arachnoïdiens; le type I: kyste arachnoïdien extradural sans compression nerveuse; Type II: Kyste arachnoïdien extradural avec compression nerveuse; Type III: Kyste arachnoïdien intradural. Dans quelques cas, le KAER peut avoir une extension intradurale [2, 3]. Dans presque tous les cas des kystes arachnoïdiens de Type I, une communication du LCS entre le kyste et l´espace arachnoïdien intrathécal par un défect dure-mérien a été rapportée [4–7]. Le cas d'un kyste arachnoïdien spinal extradural non communicant a été rapporté par LIU JK et al. [8] en 2005. L´origine exacte et la pathogénie des kystes arachnoïdiens extraduraux rachidiens (KAED) restent inconnues. L'étiopathogénie des KAED reste hypothétique et plusieurs théories ont été présentées [8]. Les KAED ont probablement une origine congénitale, et ils sont le résultat des diverticules congénitaux de la dure mère ou d'une hernie de l'arachnoïde à travers une aplasie congénitale de la dure mère [9]. Le Fourreau dural de la racine nerveuse ou la jonction de la racine et fourreau dural sont les sites les plus communs de ces défects, bien que moins souvent la ligne médiane dorsale du sac dural est aussi impliquée [4, 10]. La théorie d´une origine congénitale est corroborée par les rapports d´un syndrome familial comprenant les kystes arachnoïdiens spinaux multiples, le lymphoedème des membres inférieurs (maladie de Milroy) et distichiasis (double rangée de cils) [10–13]. Le défect de la dure mère serait dû à une anomalie structurale, d'origine congénitale, conséquence d'une défaillance de l'étanchéité des fibres collagène. Cette défaillance conduit à un allongement et une ectasie de la dure-mère. Une composante génétique dans les origines des kystes arachnoïdiens épiduraux rachidiens est également suggérée par leur association dans divers cas avec le naevus pigmenté congénital, une diastématomyélie, la sclérose en plaque, le syndrome de Marfan, une dysraphie spinale et une syringomy élie [6, 14].

Des cas de kystes arachnoïdiens spinaux qui n´ont pas clairement une origine congénitale ont aussi été rapportés. L´association de kystes arachnoïdiens spinaux avec l´arachnoïdite (source potentielle de cloisonnements arachnoïdiens), la chirurgie rachidienne et les traumatismes vertébro-médullaires a poussé quelques auteurs à suggérer que ces kystes soient susceptibles de résulter de lésions durales acquises [15]. Pareil à leur origine, Leurs mécanisme de croissance menant à la compression médullaire a été aussi un sujet de débat. Ainsi, plusieurs mécanismes ont été évoqués. La sécrétion active du liquide à partir de la paroi du kyste, L'osmose passive de l'eau, et la pression hydrostatique du LCR, ont tous été proposés comme des mécanismes possibles pour l´agrandissement du kyste. Par ailleurs, un mécanisme de ballvalve dans le pédicule de communication avec le kyste, associé à une dynamique pulsatile du LCR, aboutirait à l´expansion du kyste [5, 15]. Selon cette théorie, des poussées intermittentes de pression dans l´espace sous arachnoïdien sont communiquées au kyste et le LCR s'écoule dans la poche, Quand la pression diminue à nouveau, la compression du pédicule par le kyste inhibe l´écoulement du LCR. Selon la loi Laplace, le corps du kyste exerce une force suffisante sur le col pour fermer la communication entre le kyste et l'espace sous arachnoïdien (parce que son rayon et la tension murale sont plus grands). Ces facteurs permettent alors une nouvelle expansion parallèlement aux pulsations du LCR. Ce mécanisme deball-valve a été observé en peropératoire par Rohrer et al. [15].

Le KAER est une affection bénigne, relativement rare. Sa topographie est essentiellement dorsale (65%), s'étendant souvent sur plusieurs vertèbres. Néanmoins, la localisation lombaire et lombo-sacrée est observée dans 13% des cas, la localisation thoraco-lombaire dans 12% des cas, la localisation sacrée dans 7% et au niveau de la région cervicale dans uniquement 3% des cas [5, 11, 14]. Le KAED se développe en général chez le jeune adulte. Le KAED est habituellement de siège postérieur ou postéro-latéral. Cependant, une extension à travers un trou de conjugaison peut parfois être notée [16]. Les deux sexes peuvent être touchés, mais une prédominance masculine a été rapportée à la seconde décade de la vie. Sur le plan clinique, le KAER est le plus souvent asymptomatique et de découverte fortuite. La compression médullaire et/ou nerveuse n'a été que rarement décrite [8]. Le KAER se révèle volontiers par une parésie progressive de type spastique, d'un ou des deux membres inférieurs, associée à des paresthésies. Ailleurs, il peut s'agir de douleurs radiculaires suivies plus ou moins rapidement d'un déficit moteur. Ainsi, le tableau clinique dépend du niveau de la compression médullaire. Les symptômes peuvent fluctuer avec des périodes de émissions et d'exacerbations bien que, dans la plupart des cas, les symptômes sont en général lentement évolutifs.

Néanmoins, une révélation ou une décompensation rapide reste aussi possible. Il n'existe pas de corrélation entre la sévérité des signes cliniques et leur date d'apparition; pour les kystes thoraciques, la durée d'évolution des symptômes est plus courte que pour les kystes lombaires du fait de la différence du diamètre du canal rachidien d'une part et de la vulnérabilité de la moelle épinière par rapport aux racines de la queue de cheval [1]. Les examens complémentaires sont dominés par l'imagerie par résonance magnétique (IRM). Les radiographies simples, le scanner et le myéloscanner, encore mentionnés dans la littérature, ne présentent actuellement plus guère d’intérêt. Méthode non invasive ambulatoire, non irradiante, elle se base sur le temps de relaxation des protons soumis à un champ magnétique puissant et à des impulsions de radiofréquences déterminés. Elle permet une étude morphologique globale dans les différents plans de l'espace des différentes composantes rachidiennes : la moelle, les espaces sous arachnoïdiens et épiduraux. L'IRM reste l'examen de référence pour le diagnostic positif des KAER vu sa grande sensibilité et spécificité pour les lésions contenant du LCR. Elle montre de façon précise le siège exact, la taille, l'étendue et le caractère unique ou multiple du kyste et le degré de la compression nerveuse et l'état de la moelle épinière en regard du kyste (compression ou atrophie médullaire) [17], la présence d'éventuel ssepta qui cloisonnent le kyste.

Par ailleurs, l'IRM permet aussi de guider le choix de la voie d'abord chirurgical, d'évaluer le pronostic neurologique du patient [11, 17]. Généralement, le KAER se présente comme une masse siégeant derrière le cordon médullaire et ayant le même signal que le LCR, aussi bien sur les séquences pondérées T1 que T2. Ce signal peut cependant parfois en être différent en raison des variations de flux du liquide à l'intérieur du kyste. L'apport récent des séquences dites en écho de gradient est fondamental car de par leur excellente résolution spatiale, elles permettent de visualiser le pédicule de communication du KAER avec l'espace sous-arachnoïdien. L'injection intraveineuse de Gadolinium est utile pour éliminer les autres lésions kystiques qui peuvent prêter à confusion avec le kyste arachnoïdien épidural rachidien telles que le kyste synovial, le kyste neurentérique, le kyste dermoïde ou épidermoïde, le kyste hydatique ou aussi une tumeur purement kystique telle que le schwannome kystique et l'hémangioblastome [18]. Les caractères histologiques des kystes arachnoïdiens semblent bien définis et ne posent pas de problème diagnostique. Il s'agit d'un kyste translucide, de taille variable, dont la paroi ressemble à de l'arachnoïde épaisse. Le pertuis n'est retrouvé que dans 30% des cas rapportés par Fortuna et al. [19]. La paroi des KAER est similaire à l'arachnoïde et est constituée de lamelles de tissu collagène mature contenant des ilots de cellules arachnoïdiennes semblables au tissu arachnoïdien normal [19] Le contenu est rarement analysé, il s'agit généralement du LCR dont la teneur en protides est variable Le traitement du KAER a pour objectifs de rétablir la circulation normale du LCR, lever la compression radiculo-médullaire et éviter les récidives. La chirurgie doit permettre de rétablir le flux normal du LCR en levant la compression par le kyste. Elle doit également permettre d'effectuer des prélèvements pour un examen anatomo-pathologique permettant de confirmer un diagnostic déjà suspecté ou faire le diagnostic lorsque celui-ci n'a pas été évoqué en préopératoire. Plusieurs méthodes chirurgicales peuvent être proposées notamment une marsupialisation du kyste qui consiste à ouvrir le kyste et faire communiquer largement son contenu avec les espaces sous arachnoïdiens péri-médullaires, cependant la résection large du kyste est la méthode de choix puisque qu'elle vise à supprimer définitivement le gradient de pression entre le kyste et l'espace sous-arachnoïdien.

Pour les patients asymptomatiques, il est recommandé d'observer un traitement conservateur avec une surveillance de l'évolution de la symptomatologie clinique et des contrôles radiologiques réguliers. Concernant Les KAER symptomatiques, tous les auteurs s'accordent sur l'indication chirurgicale. Il est alors recommandé de faire l´excision complète du kyste, suivie par la ligature du pédicule communiquant le kyste avec l'espace sous-arachnoïdien et la réparation du défect dure-mérien. Il s'agit de la technique de choix afin de prévenir la réaccumulation du LCR et la récidive du kyste. Le pronostic des KAER est bon surtout s'ils sont diagnostiqués et pris en charge précocement. Néanmoins, même si la chirurgie assure de bons résultats fonctionnels, certains auteurs proposent émettent certaines réserves par rapport au pronostic en présence de certains facteurs préopératoires comme l'âge avancé, un déficit neurologique de longue durée ou une myélomalacie en IRM. Ceci peut être dû à une insuffisance vasculaire médullaire causée par la compression chronique de la moelle [20].

## Conclusion

Le KAER représente une cause rare de compression médullaire. Il peut se voir à tout âge; sa pathogénie n'est pas univoque et demeure un sujet de débat, elle peut être congénitale ou acquise. Le tableau clinique est celui d'une compression médullaire lente. L'IRM rachidienne est donc nécessaire pour asseoir le diagnostic de KAER et éliminer les autres diagnostics différentiels. Elle est donc l'examen de choix qui permet d'étudier le contenu rachidien dans son ensemble, de caractériser de façon spécifique la nature kystique du processus et d'apprécier au mieux le retentissement médullaire. Une fois le diagnostic est fait, la chirurgie est le meilleur moyen de rétablir le flux normal du LCR en levant la compression exercée par le kyste sur la moelle épinière. Le pronostic de cette pathologie histologiquement bénigne est généralement bon.
